# Urinary Proteomic Signature in Acute Decompensated Heart Failure: Advances into Molecular Pathophysiology

**DOI:** 10.3390/ijms23042344

**Published:** 2022-02-20

**Authors:** Elisa Diaz-Riera, Maísa García-Arguinzonis, Laura López, Xavier Garcia-Moll, Lina Badimon, Teresa Padro

**Affiliations:** 1 Cardiovascular-Program ICCC, Research Institute—Hospital Santa Creu i Sant Pau, IIB-Sant Pau, 08041 Barcelona, Spain; ediazr@santpau.cat (E.D.-R.); mgarciaar@santpau.cat (M.G.-A.); lbadimon@santpau.cat (L.B.); 2 Faculty of Medicine, Universtitat de Barcelona, 08036 Barcelona, Spain; 3 Cardiology Department, Hospital Santa Creu i Sant Pau, 08025 Barcelona, Spain; llopezl@santpau.cat (L.L.); xgarcia-moll@santpau.cat (X.G.-M.); 4 Centro de Investigación Biomédica en Red Cardiovascular (CIBERCV), Instituto de Salud Carlos III, 28029 Madrid, Spain; 5 Cardiovascular Research Chair, UAB, 08025 Barcelona, Spain

**Keywords:** proteomics, 2DE-MS/MS, acute decompensated heart failure, urine samples, pathophysiology

## Abstract

Acute decompensated heart failure (ADHF) is a life-threatening clinical syndrome involving multi-organ function deterioration. ADHF results from multifaceted, dysregulated pathways that remain poorly understood. Better characterization of proteins associated with heart failure decompensation is needed to gain understanding of the disease pathophysiology and support a more accurate disease phenotyping. In this study, we used an untargeted mass spectrometry (MS) proteomic approach to identify the differential urine protein signature in ADHF patients and examine its pathophysiological link to disease evolution. Urine samples were collected at hospital admission and compared with a group of healthy subjects by two-dimensional electrophoresis coupled to MALDI-TOF/TOF mass spectrometry. A differential pattern of 26 proteins (>1.5-fold change, *p* < 0.005), mostly of hepatic origin, was identified. The top four biological pathways (*p* < 0.0001; in silico analysis) were associated to the differential ADHF proteome including retinol metabolism and transport, immune response/inflammation, extracellular matrix organization, and platelet degranulation. Transthyretin (TTR) was the protein most widely represented among them. Quantitative analysis by ELISA of TTR and its binding protein, retinol-binding protein 4 (RBP4), validated the proteomic results. ROC analysis evidenced that combining RBP4 and TTR urine levels highly discriminated ADHF patients with renal dysfunction (AUC: 0.826, *p* < 0.001) and significantly predicted poor disease evolution over 18-month follow-up. In conclusion, the MS proteomic approach enabled identification of a specific urine protein signature in ADHF at hospitalization, highlighting changes in hepatic proteins such as TTR and RBP4.

## 1. Introduction

Heart failure (HF), a pathological condition characterized by the inability of the heart to pump enough blood and oxygen to support the metabolic demands of other organs [1], is nowadays a leading cause of morbidity and mortality worldwide [2]. HF is a progressive pathology with recurrent episodes of acute worsening or decompensation [3]. Hence, acute decompensated heart failure (ADHF) is a distinct clinical syndrome with a multifaceted and still incompletely understood pathophysiology, thus leaving potential for discovery of new targets to cover unmet clinical needs regarding more accurate patient risk stratification and novel preventive and therapeutic interventions. ADHF is frequently associated with diminished renal function, an important risk factor for poor outcomes [4,5]. Kidney dysfunction in HF has generally been considered a result of impaired renal blood flow in the setting of depressed cardiac function [6]. However, increasing evidence suggests a more complex and multifactorial process [7], stressing the need to gain a better understanding of the pathophysiological mechanisms linking the failing heart and the kidney.

Human body fluids containing disease-associated proteins that reflect ADHF pathophysiology might assist clinicians in early diagnosis, risk stratification, and management of the patients. More specifically, protein signals can themselves be mediators of the ADHF phenotype and represent both causal and secondary pathways leading to the development and progression (or remission) of the disease. In this respect, urine contains proteins from the kidney and urinary tract, but also from distant organs and tissues [8]. Therefore, urine proteins are particularly suitable to gain better understanding of dysfunctional processes involving these organs.

Differential proteome signatures between normal and disease states can be determined by using targeted and untargeted mass spectrometry (MS)-based proteomic approaches [9,10]. MS analysis has emerged as a powerful tool in proteomics to identify and characterize proteins and protein complexes in biological samples including blood, urine, tissues, and cells. MS can be used for high-throughput identification of proteins in complex mixtures or after protein separation by different methods including two-dimensional gel electrophoresis (2DE) [11,12]. In previous studies, we used a top-down strategy (analysis of intact protein) based on 2DE coupled to matrix-assisted laser desorption and ionization time of flight (MALDI-TOF/TOF) mass spectrometry to describe changes of the plasma proteome in early stages of acute myocardial infarction [13,14] or in isolated platelets of subjects with metabolic disorders [15]. Here, we focused on a similar 2DE-MS-based approach to characterize the differential urinary protein profile in ADHF patients at hospital admission in comparison with healthy subjects used as reference group. In silico analysis was performed to highlight the most representative differential proteins and gain better insight into the biological processes and molecular functions associated with the pathophysiology of ADHF and its association with kidney dysfunction and/or the cardiorenal syndrome.

## 2. Results

### 2.1. Clinical Characteristics of the ADHF Patient Population

The study included 67 patients who were hospitalized due to ADHF at Hospital de la Santa Creu i Sant Pau (HSCSP) in Barcelona. Baseline demographic and clinical characteristics of the studied population are given in Supplementary Table S1. ADHF patients had a median glomerular filtration rate of 61.0 (40.9–83.3) mL/min/1.73 m^2^, with 47% of patients presenting values within the pathological range (41 (31–45) mL/min/1.73m^2^). Median percentage of ventricular ejection fraction in the study population was 45 (33–58)%, with values of <40% in 27 of the 67 ADHF patients.

For the 2DE-MS studies, a subgroup representing 25% of the ADHF patients was randomly selected (76% male, 72 (69–76) years old). As shown in the Supplementary Table S2, the subgroup used in the urine proteomic studies (2DE-MS group) did not statistically differ from the total study group (Validation Group) regarding demographic characteristics (sex, age), kidney and cardiac function markers, and risk factors including hypertension and pulmonary hypertension, diabetes type 2, and dyslipidaemia. No major differences were observed concerning background medication.

### 2.2. Urine Protein Signature in ADHF Patients by 2DE Mass Spectrometry

**Figure 1:**
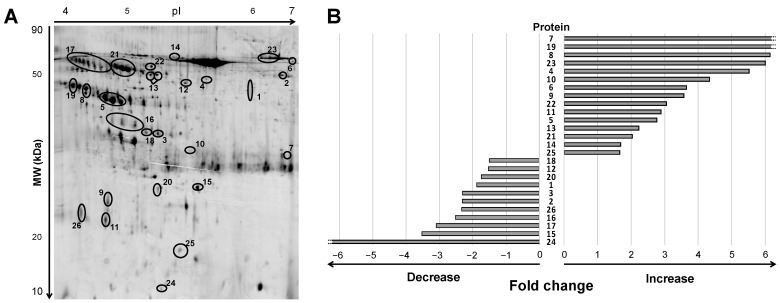
Differential protein signature by 2DE-MS in urine of ADHF patients at hospital admission. (**A**) Representative 2DE-PAGE gel of an ADHF patient depicting proteins with >1.5-fold change compared to healthy subjects. Urine proteins were separated on IPG strips (pH 4–7) in the first dimension followed by 12% SDS-PAGE in the second dimension 2D gel electrophoresis. Proteins were stained with fluorescent flamingo and images were captured with blue laser (excitation 512 nm and emission 535 nm) using a Typhoon FLA9500. (**B**) Bars refer to fold change increase/decrease labeling intensity for each protein highlighted in panel A (ID Gel spot number), in ADHF patients compared to healthy group. To note, spots identified as the same protein by MS are shown under the same ID number, and fold change in panel B was calculated by adding the individual spot intensities.

Urine samples of ADHF patients at hospital admission depicted a differential pattern of 47 protein spots compared to healthy subjects when analyzed by 2D electrophoresis (2DE) gel within a pH range of 4 to 7 and molecular weight between 10 and 80 kDa (Figure 1A and Supplementary Figure S1). After in-gel tryptic digestion of the spots, 26 non-redundant proteins were identified by mass spectrometry MALDI-TOF/TOF, as described below. Among them, 19 proteins were detected as one, single spot; two were detected as a two-spot protein, while the other five proteins showed a multi-spot pattern (Figure 1A).

Of this 19-protein subset, 15 had >1.5-fold higher levels in urine of ADHF patients than in healthy subjects, while 11 proteins presented >1.5-fold lower intensity in the ADHF group (Figure 1B). Consistency for these findings (>1.5-fold change in intensity between ADHF patients and healthy subjects across all gels) was higher than 82% for 16 proteins, 7 other proteins depicted a consistency >75%, and for 3 proteins (ID number: 12, 14, 18) it was between 58–65%.

Protein identification of the MALDI-TOF/TOF spectra was obtained using the MASCOT Server search engine on the monoisotopic mode, allowing a maximum of 100 ppm peptide tolerance and a maximum of 2 trypsin missed cleavages against the SwissProt database (see Section 4 for details). Ten of the 26 proteins were directly identified by peptide mass fingerprint (PMF-MS) with a sequence coverage ranging from 3% to 26% and MASCOT scores between 60 and 234 (reliable protein identification refers to Mascot score >56). Sixteen proteins were identified by MS/MS working on the lift mode after peptide fragmentation of the three monoisotopic peaks with higher intensity (Table 1 with additional information in Supplementary Table S3). Selected peptides had a median length of 11 (9–14) amino acids. Seven peptides presented a cysteine (C) carbamidomethylation and one peptide had a methionine (M) oxidation; both types of modifications were taken into account for the fragment mass analysis and MASCOT MS/MS ion search. Protein identification was based on the peptide with better performance after fragmentation and confirmed (*n* = 9 proteins) using results obtained from the additional selected peptides (Supplementary Table S4). Peptides 2 and 3 of seven proteins did not result in reliable protein identification and had to be discarded. MASCOT ion scores for the analyzed peptides were >50 (range: 54–102; Table 1), which was in line with a correct protein identification (MASCOT ion score threshold = 30 in MS/MS analysis).

### 2.3. Changes in the ADHF Urine Protein Signature and Relation to Kidney and Heart Function

Table 2 shows the median (Q1–Q3) values of the spot(s) intensity (arbitrary units, AU) for the 26 proteins identified by PMF-MS and MS/MS with a median fold change >1.5-fold between the ADHF and HS groups. Differences in spot intensity between groups for all proteins except for the fibrinogen chain β (*FGB*) showed Q values (FDR correction) ≤0.06, indicating a maximal expected proportion of 6% false positives among all features.

Among 26 proteins, 13 (7 decreased and 6 increased levels) showed the strongest change (>3-fold vs. HS group) in patients within the lowest tertile for the glomerular filtration rate (median MDRD-4: 33.7 (31.1–41.2) mL/min/1.73 m^2^). In addition, four of these proteins showed major changes in association with a reduced LVEF (median lower tertile <40%) (Supplementary Figure S2).

### 2.4. Functional Characteristics and Pathway Analysis of the Differential Urine Protein Signature in ADHF

**Figure 2:**
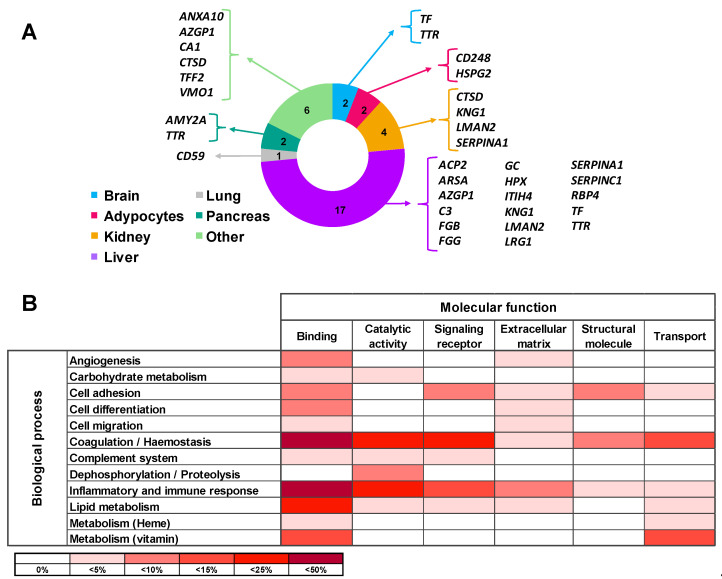
Main origin and function of the differential urinary proteins in ADHF. (**A**) Tissue origin of urinary proteins. Besides the listed organs, stomach, spleen, and salivary gland are included in the Others category. (**B**) Molecular function and biological process involving the urine differential proteins. Color intensity refers to the number of proteins involved, expressed as percentage of the total differential protein subset in urine (*n* = 26).

Tissue origin of the differential proteins in urine of ADHF patients was defined using the Genecards database. Most of these proteins (65%) are produced in the liver, with three of them being also expressed in the kidney. Differential urinary proteins in ADHF were also expressed in the pancreas, brain, adipocytes, and lung in addition to the stomach, salivary glands, and spleen. Of note, none of the ADHF differential proteins in urine had primary cardiac origin, according to the Genecards database (Figure 2A).

All 26 proteins differentially secreted in the urine of ADHF patients were subjected to the PANTHER database search for classification according to their molecular function and biological process based on Gene Ontology (GO) annotation terms. As shown in Supplementary Table S5, most of the identified proteins (85%) were involved in metabolic processes for lipids, carbohydrates, vitamins, and heme molecules (12 proteins) as well as in hemostasis (coagulation) and the complement pathway cascade (12 proteins). Other major biological processes were inflammatory and immune responses (9 proteins) and cell functions including differentiation, adhesion, and migration (6 proteins). The most common molecular functions for the differential protein pattern were protein binding (84% proteins), catalytic activity (38% proteins), and molecular transport (27%). Thirteen of the 26 proteins were related to two or more molecular functions while 11 proteins were related to several biological processes (Supplementary Table S5, Figure 2B).

**Figure 3:**
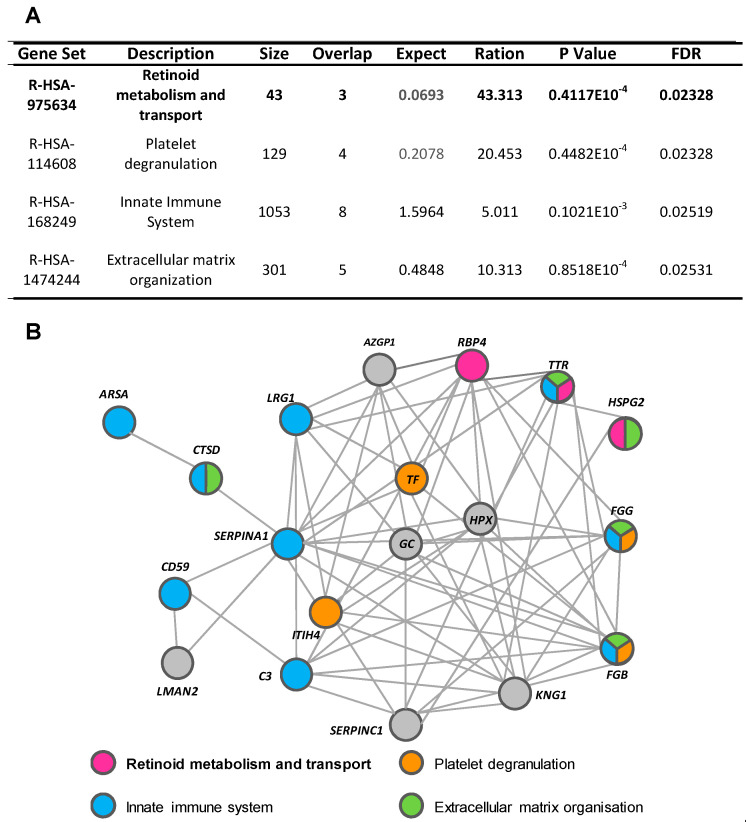
Pathways associated with identified urinary proteins. (**A**) Reactome pathways of urinary proteins of ADHF patients. (**B**) String network of urinary proteins identified in ADHF patients.

Using the WebGestalt and Reactome platforms, four biological/molecular pathways were found to be overrepresented among the 26 urine proteins with a differential detection pattern in ADHF (Q-value < 0.05; Figure 3A). These referred to retinoid metabolism and transport (R-HSA-975634), platelet degranulation (R-HSA-114608), innate immune system (R-HSA-168249), and extracellular matrix organization (R-HSA-1484244). Within the first group, three different proteins were identified: retinol-binding protein 4 (*RBP4*), transthyretin (*TTR*), and heparan sulphate proteoglycan 2 (*HSPG2* or perlecan). Four proteins were related to platelet degranulation including fibrinogen β chain (*FGB*) and γ chain (*FGG*), serotransferrin (*TF*), and inter-α-trypsin inhibitor heavy chain H4 (*ITIH4*). Moreover, nine of the proteins differentially detected in the urine of ADHF patients were associated to the innate immune system, wherein eight were increased (fibrinogen γ chains (*FGG*), α-1-antitrypsin (*A1AT*), CD59 glycoprotein (*CD59*), cathepsin D (*CTSD*), arylsulphatase A (*ARSA*), complement C3 (*C3*), transthyretin (*TTR*), and leucine-rich α-2-glycoprotein (*LRG1*) and only the fibrinogen β chain was decreased. The extracellular matrix organization pathway included five proteins, *FGG*, *TTR,* and *CTSD* with increased levels and *HSPG2* and *FGB* with decreased levels, in ADHF.

Network analysis (STRING search tool) revealed that 77% of the differential urinary proteins in ADHF are connected by direct or indirect interactions forming a single cluster, with a set of 14 proteins showing the highest number of interactions (>7 protein–protein interactions (PPI) each). From this cluster, 10 proteins participate in the top four pathways related to the differential urine signature, suggesting a close interplay between these biological processes in the physiopathology of ADHF. TTR and fibrinogen β and γ chains (*FGB* and *FGG*) were the most overrepresented proteins participating in three of the four identified pathways each (Figure 3B and Supplementary Figure S3). Both FGG and TTR showed >2-fold changes when the urine of ADHF patients and healthy subjects was compared. Of these two proteins, TTR was selected for further quantitative validation studies, based on the involvement of this protein in pathophysiological processes associated with heart disease [16,17,18], kidney disorders [19], inflammation, and malnutrition [20,21], which refer to processes related with the pathophysiology and progression of heart failure.

### 2.5. Urine and Plasma Transthyretin Levels in ADHF Patients

**Figure 4:**
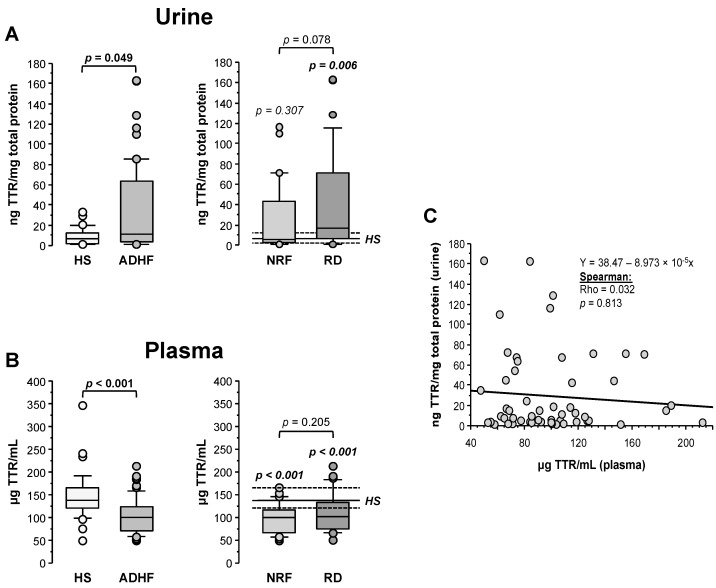
Urinary and plasma levels of transthyretin (TTR). (**A**) On the left, urinary levels of TTR obtained by immunoassay (ELISA) of healthy subjects and all ADHF patients (*n* = 67). On the right, urinary TTR of all ADHF patients with normal renal function at hospitalization (NRF, *n* = 35) and with renal dysfunction (RD, *n* = 32) at hospital admission. The *p* values in italics correspond to comparison with healthy subjects. Urine levels were normalized by total protein in urine. (**B**) TTR plasma levels of all ADHF patients and healthy subjects on the left. On the right, plasma levels of TTR of ADHF patients with normal renal function (NRF, *n* = 35) and with renal dysfunction (RD, *n* = 32) at hospital admission. The *p* values in italics correspond to the comparisons between each ADHF patient subgroup and healthy subjects. (**C**) Correlation between TTR plasma and urinary levels.

Transthyretin (TTR) was quantified by ELISA and its levels in urine were normalized by the total protein content in the sample. As shown in Figure 4A, ADHF patients (*n* = 67) showed significantly higher TTR urine levels than the healthy subject group (11.77 (3.65–64.27) vs. 6.62 (2.47–12.17) ng TTR/mg total protein, *p* = 0.049), validating the proteomic findings. Patients with renal dysfunction at hospital admission showed a trend to higher TTR levels (16.7 (6.4–70.9) TTR/mg total protein) compared to ADHF patients with normal renal function at hospital admission (5.2 (2.6–43.0) ng TTR/mg total protein, *p* = 0.078, Figure 4A).

Contrarily to urine, plasma median TTR level was significantly lower in the ADHF than in the healthy group (100.1 (71.3–123.7) vs. 136.6 (120.9–164.9) µg TTR/mL, *p* < 0.001). This difference was found regardless of the absence or presence of renal dysfunction (NRF and RD groups, *p* < 0.001 vs. HS group; Figure 4B). No differences were observed between ADHF patients with normal renal function and those with renal dysfunction at hospitalization (99.7 (66.9–115.8) µg TTR/mL vs. 101.8 (74.3–127.1) µg TTR/mL, *p* = 0.205). No correlation between TTR levels in urine and plasma was observed (Rho = 0.032, *p* = 0.813, Figure 4C), suggesting that the decrease in TTR plasma levels is not only dependent on the urinary loss but rather on a balance between tissue expression/secretion and renal filtration. Interestingly, plasma TTR levels in ADHF patients at admission inversely correlated with plasma levels of C-reactive protein (CRP), the gold standard marker for systemic inflammation (Rho = −0.303, *p* = 0.039) that was significantly increased in ADHF patients (Supplementary Figure S4). This correlation was maintained in ADHF patients with kidney dysfunction at admission (RD group; Rho = −0.432; *p* = 0.043) but not in the ADHF group with MDRD-4 levels in the physiological range (NRF group; Rho = −0.228; *p* = 0.275).

In the ADHF group, TTR levels in urine did not depend on age (Rho = −0.002; *p* = 0.986) and did not differ between sexes (men vs. women: 12.3 (3.7–67.1) vs. 9.4 (2.8–24.2) ng TTR/mg total protein; *p* = 0.561). TTR levels in the total ADHF patient group did not correlate with the LVEF (Rho = −0.050, *p* = 0.706). However, as shown in Supplementary Table S6, among ADHF patients with normal renal function (NRF group), those with reduced LVEF (<40%) had a TTR loss in urine >3-fold higher than patients with preserved LVEF (12.3 (3.3–64.1) vs. 4.1 (1.6–5.0) ng TTR/mg total protein; *p* = 0.049). This pattern was not observed in ADHF patients with renal dysfunction (RD group) at hospital admission, who presented high urine TTR loss regardless of the LVEF condition (reduced 12.0 (3.3–54.5) vs. preserved 15.1 (7.2–70.7) ng TTR/mg total protein, *p* = 0.363). In addition, urinary TTR levels did not differ between patients with and without a clinical history of ischemia and presence of cardiovascular risk factors and clinical characteristics such as atrial fibrillation and pulmonary hypertension, either in the total ADHF group or when the subgroups without and with renal dysfunction at admission were separately considered (see Supplementary Table S6).

### 2.6. Changes in Urine RBP4 Directly Correlate with TTR and Are Related to Renal Function

**Figure 5:**
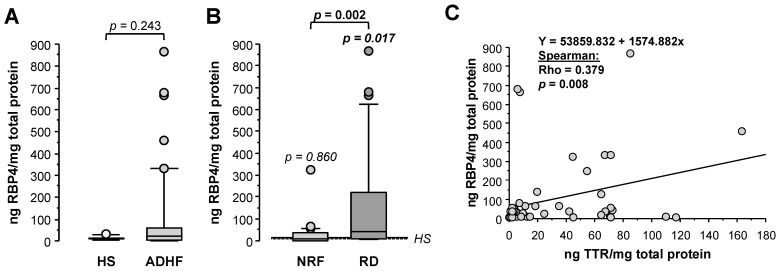
Urinary levels of retinol-binding protein 4 (RBP4). (**A**) Urinary levels of RBP4 obtained by immunoassay (ELISA) of all ADHF patients (*n* = 67) at hospital admission and healthy subjects. (**B**) Urinary levels of RBP4 of ADHF patients with normal renal function (NRF, *n* = 35) and with renal dysfunction (RD, *n* = 32) at hospital admission. The *p* values in italics correspond to comparison with healthy subjects. (**C**) Regression line of urinary RBP4 and TTR levels of ADHF patients at hospital admission.

Retinol-binding protein 4 (RBP4), unlike results obtained by proteomics, showed 2-fold higher loss in urine for ADHF patients when compared with healthy subjects (HS), although differences did not achieve statistical significance (22.8 (3.8–60.9) vs. 11.9 (7.8–14.0) ng RBP4/mg total protein, *p* = 0.244, Figure 5A). Of note, ADHF patients with renal dysfunction (RD) at hospital admission had >4-fold higher RBP4 urine levels than those presenting normal renal function, who otherwise had urine values within the normal range (RD vs. NRF groups: 40.9 (9.5–248.7) vs. 9.7 (2.0–34.9) ng RBP4/mg total protein, *p* = 0.002, Figure 5B). A significant positive correlation was found between urinary levels of RBP4 and its transporter TTR in ADHF patients (Figure 5C).

### 2.7. RBP4 and TTR Levels in Urine at Hospital Admission Relate with Disease Evolution within 18-Month Follow-Up

**Figure 6:**
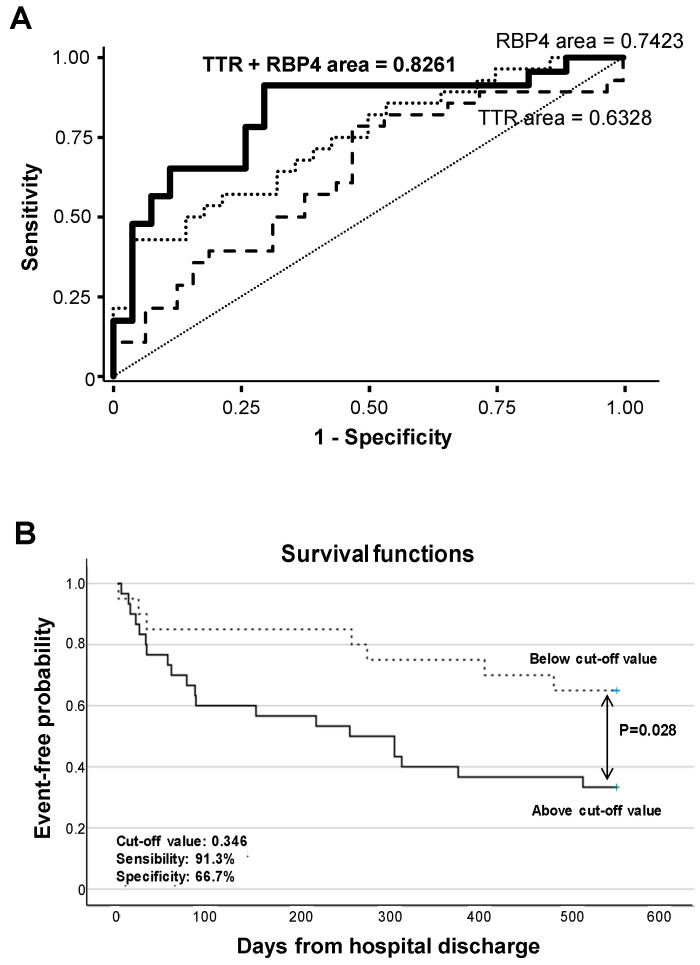
Discrimination and survival analyses of TTR and RBP4 levels. (**A**) ROC analysis of urinary TTR and RBP4 for the discrimination of ADHF by their glomerular filtration rate. (**B**) Kaplan–Meier curve analysis. Only the first major adverse clinical event during follow-up (28 rehospitalizations due to heart and/or kidney decompensation, 4 heart transplants, and 8 patient deaths) was considered for calculation of event-free probability.

Receiver operating characteristic (ROC) curve analysis evidenced that RBP4 urine levels significantly differentiate ADHF by their glomerular filtration rate (MDRD-4 values), with an AUC of 0.742 (95% CI (0.614–0.870), *p* < 0.001), with the cutoff value of 37.0 ng/mg total protein (57.1% sensitivity and 78.6% specificity) being the urine concentration that better discriminated patients with and without pathological glomerular filtration (MDRD-4 < 60 mL/min/1.73m^2^) at hospital admission. In addition, ROC analysis showed that combining RBP4 and TTR urine levels resulted in higher C-statistic values (AUC: 0.826 (0.705–0.947), *p* < 0.001) for discriminating ADHF patients according MDRD-4 levels (Figure 6A, Supplementary Table S7).

Within the 18-month follow-up after hospital discharge, 59% of the ADHF patients presented major clinical outcomes, which included rehospitalization (28 patients) due to heart and/or kidney decompensation, heart transplant (4 patients), and death (12 patients).

Kaplan–Meier analysis evidenced that values above a predicted probability of 0.346 (specificity 66.7%, sensitivity 91.3%) calculated from the AUC for combined urine levels of RBP4 and TTR at hospital admission associated with worse disease evolution and earlier presentation of major adverse events (cardiac and/or renal rehospitalization, heart transplant, or death, *p* = 0.028, Figure 6B) during the 18 months’ evolution follow-up.

## 3. Discussion

Acute decompensated heart failure (ADHF) is a complex clinical condition that may affect different organs and involve several pathophysiological mechanisms. Until now, the underlying biological processes have not been completely elucidated, emphasizing the need for a deeper understanding of the molecular functions and pathways associated to the ADHF pathophysiology. To address this problem, we carried out a discovery hypothesis-free study using 2DE coupled to MALDI-TOF/TOF mass spectrometry (2DE-MS) aimed to unravel a disease-specific differential proteomic profile in urine of ADHF patients at hospital admission and its potential link to pathophysiology.

In recent years, urine has become a promising biospecimen in clinical proteomics. Urine represents a combination of both blood ultrafiltrates and local secretion from kidney-specific cells and tubules, therefore reflecting systemic and renal diseases [22]. Moreover, in the absence of homeostatic regulation, the changes in urine proteins may detect small and early pathological changes [23]. Additionally, urine sampling has many advantages compared to blood including the availability of larger and more recurrent volumes without causing discomfort to the patient.

Here, by investigating the urine proteomic pattern of ADHF patients at hospital admission, we identified a protein signature of 26 unique proteins associated to the acute decompensation of heart failure that could characterize the pathophysiological changes in ADHF patients. Overall, the differential pattern refers to proteins acting through molecular functions such as catalytic activity, signaling receptor binding and transport, and being involved in various biological processes including metabolism of lipids, vitamins, carbohydrates and heme-components, hemostatic and complement systems, and immune and inflammatory responses.

Of the 26 proteins with differential urine detection levels in ADHF, 17 were mainly of hepatic origin and, among them, 11 proteins showed the highest changes in the urine of ADHF patients with glomerular filtration below the pathological threshold (MDRD-4 <60 mL/min/1.73m^2^), which might relate to the concept of a pathological interaction among heart, kidney, and liver in heart failure patients leading to multi-organ deterioration and unfavorable disease evolution [24,25,26]. In this respect, Kawahira et al. [27] recently reported the prognostic value of impaired hepato-renal function in patients hospitalized for acute decompensated heart failure by combining the MELD-XI score, which includes data on bilirubin and creatinine reflecting liver and kidney function and the FIB-4 index that assesses liver fibrosis [28]. Indeed, cardiac dysfunction, especially in ADHF, can cause, in addition to elevated venous pressure, reduced liver blood flow and arterial flow, leading to hepatocyte atrophy, edema of the peripheral area, and liver stiffness [27,29]. Extending these clinical observations, in our study, leucine rich α-2-glycoprotein (*LRG1*) was near 100-fold higher in urine of ADHF patients. *LRG1* is a hepatic protein that is known to regulate endothelial TGFβ signaling, a major factor in many progressive fibrotic diseases [30]. The potential relevance of *LRG1* in cardiac fibrosis has recently been suggested in a mice experimental model of chronic pressure overload-induced heart failure [31]. Additionally, zinc-α2-glycoprotein (*AZGP1*), with 3–5-fold higher loss in urine of ADHF patients, might contribute to pathological tissue remodeling and disease progression. Thus, Sörensen-Zender et al. [32], using mice genetically deficient in *AZGP1,* described this protein to be involved in negative regulation of fibrosis through a TGFβ-mediated mechanism.

In silico data analysis led to several other noteworthy observations that might contribute to gaining better understanding of the molecular events and pathological mechanisms participating in ADHF. Thus, an interesting insight that merged from using search tools such as WebGestalt, Reactome, and STRING was that the differential urinary protein subset fell into the top four categories of signal networks, including innate immune system and inflammation, platelet degranulation, extracellular matrix organization, and retinoid metabolism and transport. Furthermore, our study evidenced that 19 of the urine differential proteins participating in the four categories were interacting in a single network cluster, suggesting a close interplay among various biological pathways in association to the ADHF pathology. Cardiac decompensation accompanying acute heart failure (AHF) episodes has been related to systemic inflammatory responses, supported by elevated levels of high-sensitivity C-reactive protein (CRP), primarily reflecting innate immunity [33]. Additionally, inteleukin-1 beta (IL-1β), an end product resulting from the activation of the multimeric protein complex inflammasome, was associated with increased disease severity and risk of death in patients with acute decompensated heart failure [34].

Up to now, however, signaling pathways and molecules involved in this immune activation and systemic inflammatory response in ADHF were not known. Herein, extending the previous findings, we identified changes in urine levels of nine proteins linked to the innate immune system including fibrinogen, complement C3, glycoprotein CD59, α-1-antitrypsin (*SERPINA1*), cathepsin D (*CTSD*), arylsulphatase A (*ARSA*), leucine-rich α-2-glycoprotein (*LRG1*), and transthyretin (*TTR*). As such, cathepsin D, a major lysosomal protease involved in protein degradation and proteolytic activation of hormones and growth factors, is increasingly recognized for its involvement in inflammatory responses [35]. High serum levels of cathepsin D have been shown to associate with new-onset HF following ST segment elevation acute myocardial infarction [36]. Similarly, results from the BIOSTAT-CHF study (BIOlogy Study to TAilored Treatment in Chronic Heart Failure) evidenced that higher circulating cathepsin D levels correlate with more severe disease and higher rates of mortality and hospitalization in HF [37]. In addition, recent microarray gene expression data of peripheral blood mononuclear cells and single-cell RNA sequencing data of cardiac macrophages have associated the upregulation of CTSD expression with a higher risk of developing a heart failure event within 6 months after suffering an acute myocardial infarction [38]. Results of a liquid chromatography (LC)–MS analysis of platelets in a dog model of acute congestive HF identified cathepsin D among 14 proteins with differential expression level related to the disease presentation [10]. Interestingly, platelet degranulation due to activation processes was among the four most represented biological functions related to the ADHF urine differential proteome in our study. Extrapolating from studies in ischemic heart disease [39], platelet activation has been suggested as a link between HF decompensation and troponin elevation, which otherwise accounts for higher rates of in-hospital mortality and post-discharge morbidity and mortality [40].

Reactome pathway analysis evidenced fibrinogen (beta and gamma chains) and transthyretin (TTR) as the best represented proteins, each one of them participating in three of the top four pathways and converging into key biological functions relevant for acute decompensated HF, such as inflammation and extracellular matrix (ECM) remodeling. Among them, only the fibrinogen gamma chain (FGG) and transthyretin showed >2-fold changes compared to healthy subjects. Both FGG and TTR are multifaceted proteins of hepatic origin, participating in mechanistic processes relevant in maintaining the organ homeostasis.

In the present study, as proof of concept, to validate the potential translational value of the differential proteomic signature identified by 2DE-MS in ADHF patients, we further analyzed levels of TTR and its binding ligand RBP4 using commercially available ELISA assays and compared with the clinical characteristics of the patients at hospital admission and disease progression within 18 months for follow-up after hospital discharge.

TTR triggers amyloid processes, and TTR amyloid cardiomyopathy is increasingly being recognized in the clinical setting as a possible heart failure origin [41]. Additionally, TTR, along with serotransferrin (TF), is involved in the acute inflammatory response in increased chronic inflammation [9]. More importantly, TTR is a crucial protein involved in the transport of retinol to peripheral tissues after forming a homotetramer complex with the retinol-specific carrier RBP4. At the molecular level, RBP4 promotes inflammatory damage to cardiac myocytes by Toll-like receptor 4 activation [42]. Until now, data on the role of RBP4 in patients with HF were scarce and with apparently controversial findings [43,44]. In addition, TTR is one of the main carriers of thyroxine (T4), a thyroid hormone associated with blood pressure and LVEF in ADHF during hospitalization [45]. Indeed, thyroid hormones have central regulatory actions in the cardiovascular system and patients with even mildly altered thyroid function have a worse prognosis in heart disease, particularly heart failure [46].

As identified by 2DE-MS, quantitative analysis with specific immunoassays evidenced higher loss of TTR in urine of ADHF patients, the difference being more evident in the group of patients with a deficient glomerular filtration according the MDRD-4 value. Interestingly, ADHF patients showed a lower median TTR level in plasma compared to the concentration range in healthy subjects, but this decrease was irrespective of the kidney function and did not correlate with the level of TTR in urine, suggesting that TTR values in plasma result from a dynamic balance between synthesis in the liver and secretion through the kidney. In agreement with the current findings, our group had previously evidenced significantly lower TTR levels in plasma of patients with an acute new-onset myocardial infarction (AMI) compared to healthy subjects [47]. Of note, plasma levels of the inflammatory marker CRP were significantly increased in ADHF patients at hospitalization and inversely correlated with plasma TTR. Similarly, the reported TTR decrease in AMI was especially evident in patients having CRP levels >3 mg/L at the moment of admission [47]. These results strongly suggest that the increased inflammatory background in ADHF patients might account for the decrease in TTR plasma levels in ADHF, beyond changes in glomerular filtration. Supporting this view, TTR plasma decrease in ADHF patients was found regardless of the kidney functional condition.

Inflammation is a major feature in heart failure [48], and the hepatic synthesis of transthyretin has been reported to be negatively regulated by inflammatory-related mechanisms and inflammatory cytokines such as interleukin 6 (IL-6) in a process dependent on the IL-6 nuclear factor or its homologous C/EBP nuclear factor [49]. Indeed, TTR is a negative acute-phase protein, and patients with severe sepsis often have very low TTR concentrations [50]. Recently, low plasma transthyretin concentration has been shown to associate with incident heart failure in the general population [51], and it is suggested that low plasma TTR levels could be a biomarker of transthyretin tetramer instability [52]. Increasing evidence supports the view that TTR tetramers, under unbalanced homeostatic conditions, dissociate in misfolded monomers that tend to aggregate and fibrillate and, after infiltrating the cardiac extracellular matrix, induce oxidative stress and mitochondrial damage and increase cardiac wall thickness and diastolic dysfunction [53]. Further studies are needed to better characterize the molecular mechanisms relating inflammation with TTR expression and/or its structural conformation in heart failure and the potential relevance of TTR monomers on the ADHF pathophysiology.

In the urine of ADHF patients, TTR was detected as a single spot of 15 kDa, which corresponds to its monomeric form. Due to the correlation between TTR and RBP4 levels in urine of ADHF patients, it is conceivable that the monomeric form was already abundantly present in the circulating blood since only TTR tetramers bind RBP4 and protect this low-molecular-weight transport protein (21 kDa) from being filtrated through the glomerulus [54]. We could not exclude, however, that TTR-non-related RBP4 forms also account for the increased urinary levels of this protein in ADHF. In this respect, Perduca et al. [55], by high-resolution, three-dimensional structure analysis of urine-purified RBP4, identified a second RBP4 form with high binding capacity to fatty acids but not to retinol and with low affinity for TTR. This complex RBP4 fatty acid form was only identified in urine in the presence of a glomerulopathy [55]. Accordingly, this RBP4 fatty acid complex form might account for the high increase in urinary RBP4 in ADHF patients with renal dysfunction compared with those with normal renal dysfunction at hospitalization and the healthy group. Further studies are needed to understand the pathophysiological relevance of these RBP4 fatty acid complex forms in ADHF.

To the best of our knowledge, the concomitant loss of TTR and RBP4 in urine during the early phase (hospital admission) of acute heart failure decompensation and their power when combined to identify patients with worse disease evolution and prognosis within an 18-month follow-up after hospital discharge have not been previously reported in the clinical setting of ADHF. Indeed, the Kaplan–Meier curve analysis revealed a potential implication of increased urinary loss of TTR and RBP4 in the disease progression and presentation of major adverse events.

## 4. Materials and Methods

### 4.1. Study Population and Study Design

This study included 67 patients (men and women over 18 years old; 71 (65–77) years old, 67% men) who were hospitalized due to ADHF between February 2017 and March 2020 at Hospital de la Santa Creu i Sant Pau (HSCSP) in Barcelona. Hospitalized ADHF patients were distributed into two different groups depending on their kidney function at admission. Renal function was given as MDRD-4 (mL/min/1.73m^2^), which is a serum creatinine-based estimation obtained using the clinical data of the patients [56]. Levels below 60 mL/min/1.73m^2^ were considered pathological. Two groups were formed: (1) ADHF patients with renal dysfunction at hospital admission (RD, *n* = 32) and (2) ADHF patients with normal renal function at hospital admission (NRF, *n* = 35) (Supplementary Table S1). After hospital discharge, ADHF patient were followed for 18 months and the adverse clinical outcomes (rehospitalization due to decompensation, heart transplant, and/or death) were registered. Twenty-eight patients required another hospitalization, 4 required heart transplants, and 12 patients died. A group of healthy subjects (HS, *n* = 35, 50.5 (48.0–54.5) years) served to establish the normal range in the urine of the identified proteins.

The Ethics Committee of the Santa Creu i Sant Pau Hospital in Barcelona, Spain, approved this study, and it was performed according to principles of Helsinki’s Declaration. All patients signed an informed consent prior to being included in the study. Patients undergoing chemotherapy, who were pregnant or had post-delivery ischemic heart syndrome in women, or had other causes of acute episode (myocardial infarction, myocarditis, or toxic etiology) were excluded from the study. Medications were not considered as exclusion criteria except those drugs required in oncological treatment (and patients who were already excluded).

### 4.2. Biological Samples

Urine and blood samples were collected at hospital admission. Urine samples were centrifuged to precipitate debris, aliquoted, and stored at −80 °C until further analysis. Urine samples for the HS reference group were collected in the morning, and samples were processed as described for the ADHF patients. Level of total protein in urine was analyzed in a Clima MC-15 analyzer using the specific Gernon kit (RAL S.A., Barcelona, Spain), as described by the providers.

Venous blood was drawn, after a 10–14-h fasting, from the cubital vein without tourniquet using a 20-gauge needle for all patients. All samples were processed identically within the first 2 h after extraction. Serum was aliquoted and stored at −80 °C.

Blood creatinine (Jaffe reaction), NT-proBNP (electroquimioluminiscence), urea (kinetic urease), and hemoglobin were analyzed by standard laboratory methods as part of the patients’ routine analyses. Glomerular filtrate was calculated using the MDRD-4 algorithm that includes a patient’s plasma creatinine levels, age, sex, and race [56].

### 4.3. Two-Dimmensional Gel Electrophoresis and MALDI-TOF/TOF MS

Analysis of differential protein patterns was performed by two-dimensional gel electrophoresis (2DE) coupled to mass spectrometry, as previously described [47,57,58].

Protein loads of 100 μg (analytical gels) and 300 μg (preparative gels) of the urea/thiourea/chaps urine extracts were applied to 17-cm dry strips (ReadyStrips IPG strips, pH 4–7 linear range; BioRad, San Diego, CA, USA) using the PROTEAN i12 IEF system (Bio-Rad, San Diego, CA, USA) for the first dimension, as previously described by our group [57,58]. The second dimension was resolved in 12% SDS-PAGE. Gels were fixed for 2 h (40% ethanol, 10% acetic acid) and developed with Flamingo (Bio-Rad, San Diego, CA, USA) for protein fluorescent staining using Typhoon 9500 with excitation wavelength at 512 nm, emission light wavelength of 535 nm, and an LPB filter. Protein spot quantification and analysis for differences between gels were performed using PDQuest analysis software (Bio-Rad, San Diego, CA, USA). Each spot was assigned a relative value (AU) that corresponded to the single spot volume compared to the volume of all spots in the gel, following background extraction and normalization between gels, as previously reported [57]. This software specifically analyzes differences in protein patterns, in which a master gel is created wherein all gels are included and used to compare with each individual sample.

Urine samples (4 mL) of ADHF patients and healthy controls were concentrated and desalted by centrifugation (3220 g, 30 min, and 10 °C) using 3-kDa cutoff filter devices (Amicon Ultra-4, Millipore, Burlington, MA, USA) and 100 mM Tris-HCl, pH 7.6. A final volume of 1 mL was obtained and depleted of albumin and IgGs using the ProteoExtract Albumin/IgG Removal Kit (Calbiochem, San Diego, CA, USA), as reported by the providers. Thereafter, a sample buffer was exchanged to a urea-containing buffer (7M urea, 2M thiourea, 2% CHAPS) by centrifugation with the 3-kDa cutoff filter devices (3220 g, at room temperature) until a final volume of 400 µL was obtained. Protein concentration in urine extracts was measured with 2D-Quant Kit (GE Healthcare, Chicago, IL, USA).

Proteins were identified after in-gel tryptic digestion and extraction of peptides from the gel pieces by matrix-assisted laser desorption/ionization time of flight (MALDI-TOF) using an AutoFlex III Smart beam MALDI-TOF/TOF (BrukerDaltonics, Billerica, MA, USA), as previously described [57]. Briefly, 1-mm^2^ gel pieces were washed first with 25 mM ammonium bicarbonate for 20 min, with 25 mM ammonium bicarbonate/50% acetonitrile (3 times for 20 min), and finally dried with 100% acetonitrile. Then, gel pieces were rehydrated with 0.2 ng/µL of trypsin Gold (Promega) in 25 nM ammonium bicarbonate and incubated overnight at 30 °C. Trypsin activity was stopped by the addition of acetonitrile for 15 min at 37 °C, and peptides were extracted with 0.2% TFA after 30 min at room temperature. Peptides were concentrated using µC18-Zip Tips (Merck-Millipore) according to manufacturer instructions. Samples and calibrants were mixed 1:1 with an alpha-Cyano-4-hydroxycinnamic acid (HCCA) matrix (0.7 mg/mL) and were applied to Anchor Chip plates (BrukerDaltonics, Billerica, MA, USA).

Spectra were acquired with flexControl on reflectron mode (mass range m/z 850–4000; reflectron 1, 21.06 kV; reflectron 2, 9.77 kV; ion source 1 voltage, 19 kV; ion source 2, 16.5 kV; detection gain, 2.37×) with an average of 3500 added shots at a frequency of 200 Hz. Samples were processed with flexAnalysis (version 3.0, Bruker Daltonics, Billerica, MA, USA) considering a signal-to-noise ratio >3, applying statistical calibration and eliminating background peaks. After processing, spectra were sent to the interface BioTools (version 3.2, Bruker Daltonics, Billerica, MA, USA), and MASCOT search on Swiss-Prot 57.15 database was performed (taxonomy, Homo sapiens; mass tolerance, 50 to 100; up to two trypsin missed cleavages; global modification: carbamidomethyl (C); variable modification: oxidation (M)). Identification was carried out by peptide mass fingerprinting (PMF) where a MASCOT score >56 and at least five matched peptides were accepted. Confirmation of the identified protein was performed by peptide fragmentation working on the LIFT mode (MS/MS) [57,58].

### 4.4. In Silico Analysis

The major bioinformatics tool GO was used to identify the function of genes and gene products of Homo sapiens. Through the WEB-based GEne SeT AnaLysis Toolkit (WebGestalt), the GO analysis was performed using the PANTHER (Protein ANalysis THrough Evolutionary Relationships) classification database [59], and the pathway analysis was performed using Reactome [60].

STRING, an online, freely available software tool, was used to establish the PPI network [61], and all the cutoff points were combined to analyze the topology property of networks.

### 4.5. Enzyme-Linked Immunosorbent Assays

Identified proteins in urine and serum were quantified by enzyme-linked immunosorbent assay (ELISA) using the following kits. Transthyretin (TTR) levels in urine and plasma were analyzed using a Prealbumin (Transthyretin) ELISA kit (K6331, Immundiagnostik, Bensheim, Germany), with intra-assay precision of 3.4%, and 5.6% for inter-assay precision. Quantification was performed using only samples where TTR was detectable (90% of total). RBP4 urinary levels were analyzed using a Human Retinol Binding Protein 4 ELISA kit (ab196264, Abcam, Cambridge, UK), with intra-assay precision 5.1% and inter-assay precision of 8.9%. Concentrations of the urinary proteins (obtained by ELISA) were normalized with urine total protein content, measured in a Clima MC-15 analyzer using the specific Gernon kit to avoid any possible bias due to interindividual differences in protein secretion.

### 4.6. Statistical Analysis

Data are expressed as median and interquartile range (IQR). The *n* indicates the number of subjects tested. The normal distribution was determined via Kolmogorov–Smirnov test. Statistical differences between groups for non-normally distributed continuous variables were analyzed by non-parametric tests, including Mann–Whitney or Kruskal–Wallis tests. Frequencies of categorical variables were compared by Fisher exact and Chi-square analyses. Correlations between variables were determined using Spearman rank correlation and pictured by single regression models. Due to the exploratory character of this proteomic study, determination of the sample size was based on past experience with similar studies [57]. The variability observed in the data (ADHF patients with and without renal dysfunction at hospital admission) from the proteomic studies served to guide the sample size in the quantitative analysis (ELISA method). Sample size was validated using the JavaScript-based method for simple power and sample size calculation when two independent groups are compared, which are provided in http://www.stat.ubc.ca/~rollin/stats/ssize/n2.html (accessed on 29 January 2022) [57]. Based on the mean urine TTR values of ADHF patients and HS and the pooled standard deviation of both groups (ADHF and HS), a sample size >32 gave a study power of >0.75 (type I error = 0.05, two-sided test).

Receiver operating characteristic (ROC) curve estimations and their corresponding C statistics (area under the curve (AUC) with their 95% CI) were calculated to determine the power to discriminate ADHF patients according to kidney function. Kaplan–Meier survival (free of adverse outcomes) analysis was performed after including the study variables in a logistic rank analysis to evaluate the value of the studied parameters for predicting disease progression (adverse outcome incidence) in ADHF patients.

Adjustments for multiple testing in the discovery proteomic study were performed by the false-discovery rate (FDR) using a two-stage, sharpened method described by Benjamini et al. [62]. The results of the calculated adjusted q values (FDR adjusted) indicated the probability of false positives for the identified proteins considered to be significant. Here, it refers to q values < 0.06, which represents more than 94% truly positive for differentially expressed urinary proteins.

Statistical analysis was performed using Stata v15 (SAS Institute, Cary, NC, USA) and SPSS v26 (IBM Corp, Armonk, NY). A *p* value ≤ 0.05 was considered statistically significant.

## 5. Conclusions

In the present study, by applying a proteomic approach based on 2D electrophoresis coupled with MALDI-TOF/TOF MS, we found a differential protein signature in the urine of patients presenting with acute decompensated heart failure at the moment of hospital admission. The 26-protein pattern highlights the complexity of ADHF pathophysiology with coordinated changes in proteins involved in molecular functions and biological processes related to the disease progression. Thus, we reported an increased urinary loss of proteins such as TTR and RBP4, which might have harmful impact on the disease evolution, resulting in higher risk for adverse outcomes after hospital discharge. Future studies are now warranted in larger populations to validate the relevance of the observed changes in TTR and RBP4 for the ADHF pathophysiology.

## Figures and Tables

**Table 1:** Mass spectrometry characteristics of identified proteins in urine of ADHF patients.

#	Protein name	Gene name	Swiss Prot number	Up/down regulation	Experimental pI	Molecular weight (KDa)	MS or MS/MS	MASCOT Score	Coverage
1	Lysosomal acid phosphatase	*ACP2*	P11117	↓	5.80	45.5	MS	63	7
2	Pancreatic α-amylase	*AMY2A*	P04746	↓	6.45	51.6	MS/MS	61	-
3	Annexin A10	*ANXA10*	Q9UJ72	↓	5.20	35.2	MS	60	19
4	Arylsulfatase A	*ARSA*	P15289	↑	5.50	49.6	MS	67	10
5	Zinc-α-2-glycoprotein	*AZGP1*	P25311	↑	4.8-5.1	41.6-43.5	MS	66	11
6	Complement C3	*C3*	P01024	↑	6.75	54.8	MS	62	3
7	Carbonic anhydrase 1	*CA1*	P00915	↑	6.70	30.1	MS/MS	71	-
8	Endosialin	*CD248*	Q9HCU0	↑	4.70	44.6-45.6	MS/MS	54	
9	CD59 glycoprotein	*CD59*	P13987	↑	4.90	22.7	MS/MS	82	-
10	Cathepsin D	*CTSD*	P07339	↑	5.40	31.2	MS/MS	58	-
11	Fibrinogen β-chain	*FGB*	P02675	↓	4.90	18.7-19.8	MS/MS	55	
12	Fibrinogen γ-chain	*FGG*	P02679	↑	5.30-5.35	48.0-48.2	MS/MS	55	-
13	Vitamin D binding protein	*GC*	P02774	↑	5.20	50.5	MS	94	17
14	Hemopexin	*HPX*	P02790	↑	5.30-5.35	55.4	MS/MS	70	
15	Basement membrane-specific heparan sulfate proteoglycan core protein	*HSPG2*	P98160	↓	5.40	24.9	MS/MS	102	-
16	Inter-alpha-trypsin inhibitor heavy chain H4	*ITIH4*	Q14624	↓	4.9-5.1	36.6-37.2	MS	74	10
17	Kininogen-1	*KNG1*	P01042	↓	4.7-4.9	50.5-53.3	MS	63	8
18	Vesicular integral-membrane protein VIP36	*LMAN2*	Q12907	↓	5.20	35.2	MS/MS	60	-
19	Leucine-rich alpha-2-glycoprotein	*LRG1*	P02750	↑	4.60	47.4	MS/MS	79	-
20	Retinol binding protein	*RBP4*	P02753	↓	5.20	24.7-25.2	MS/MS	66	-
21	Alpha-1-antitrypsin	*SERPINA1*	P01009	↑	5.00-5.10	51.4-52.2	MS	130	19
22	Antithrombin III	*SERPINC1*	P01008	↑	5.20	52.6	MS/MS	78	-
23	Serotransferrin	*TF*	P02787	↑	6.00-6.40	56.5	MS	234	26
24	Trefoil factor 2	*TFF2*	Q03403	↓	5.20	11.1	MS/MS	83	-
25	Transthyretin	*TTR*	P02766	↑	5.30	15.9	MS/MS	61	-
26	Vitelline membrane outer layer protein 1 homolog	*VMO1*	Q7Z5L0	↓	4.65	21.2	MS/MS	87	-

#: 2DE—Gel ID number; pI: isoelectric point; MS: mass spectrometry. MS indicates MALDI-TOF while MS/MS refers to MALDI-TOF/TOF.

**Table 2:** Urinary spot volumes (AU) of the differential protein signature (2DE-MS) in urine of ADHF patients at hospital admission.

Protein ^a^	Gene	HS (N=6)	ADHF (N=17)	*p* Value ^b^	Q Value ^c^
1	*ACP2*	0.89 [0.51–1.10]	0.37 [0.24–0.42]	0.049	0.046
2	*AMY2A*	1.07 [0.92–1.10]	0.47 [0.31–1.18]	0.089	0.060
3	*ANXA10*	0.55 [0.45–0.85]	0.33 [0.02–0.54]	0.077	0.056
4	*ARSA*	0.02 [0.001–0.04]	0.10 [0.06–0.20]	0.017	0.028
5	*AZGP1*	2.47 [1.61–2.81]	8.08 [4.92–12.94]	0.001	0.010
6	*C3*	0.24 [0.22–0.81]	0.89 [0.36–1.41]	0.083	0.058
7	*CA1*	0.002 [0.001–0.05]	0.27 [0.18–0.79]	0.010	0.027
8	*CD248*	0.18 [0.15–0.19]	1.12 [0.52–1.65]	0.003	0.013
9	*CD59*	0.47 [0.17–1.12]	1.68 [1.07–2.39]	0.027	0.038
10	*CTSD*	0.21 [0.16–0.33]	0.90 [0.21–1.36]	0.052	0.046
11	*FGB*	3.23 [2.60–3.87]	2.11 [1.57–2.78]	0.210	0.136
12	*FGG*	0.19 [0.02–0.28]	0.58 [0.15–1.75]	0.062	0.051
13	*GC*	0.77 [0.66–0.98]	1.71 [0.87–2.55]	0.042	0.044
14	*HPX*	1.00 [0.63–1.41]	2.05 [1.00–2.97]	0.048	0.046
15	*HSPG2*	3.96 [2.27–6.47]	1.13 [0.67–2.40]	0.015	0.028
16	*ITIH4*	10.66 [7.82–14.06]	4.25 [2.48–8.04]	0.006	0.021
17	*KNG1*	26.14 [18.40–35.01]	8.73 [3.82–16.11]	0.003	0.013
18	*LMAN2*	0.34 [0.28–0.48]	0.22 [0.07–0.35]	0.077	0.056
19	*LRG1*	0.09 [0.07–0.10]	0.87 [0.35–1.26]	0.014	0.028
20	*RBP4*	1.56 [1.41–1.68]	0.86 [0.71–1.16]	0.033	0.040
21	*SERPINA1*	1.58 [1.23–1.83]	4.53 [3.13–7.41]	0.013	0.028
22	*SERPINC1*	0.15 [0.11–0.19]	0.47 [0.20–0.63]	0.034	0.040
23	*TF*	1.22 [0.97–1.81]	7.31 [4.56–7.78]	0.001	0.010
24	*TFF2*	0.36 [0.002–0.41]	0.002 [0.001–0.06]	0.064	0.051
25	*TTR*	0.40 [0.39–0.67]	0.88 [0.66–1.45]	0.031	0.040
26	*VMO1*	4.03 [2.60–4.57]	1.74 [1.20–3.17]	0.023	0.035

^a^ 2DE—Gel ID number. Values are given as median [Q1–Q3]; ^b^ *p* values obtained by the Mann-Whitney test; ^c^ Q values obtained after FDR correction.

## Data Availability

The data presented in this study are available in supplementary material.

## Supplementary Materials

The following are available online at https://www.mdpi.com/article/10.3390/ijms23042344/s1.
