# Supplementary material for: Urinary Proteomic Signature in Acute Decompensated Heart Failure: Advances into Molecular Pathophysiology
Source: Int J Mol Sci. 2022 Feb 20;23(4):2344. doi: 10.3390/ijms23042344 (PMC8875709; doi:10.3390/ijms23042344)
Supplement: Supplementary file 1 [file ijms-23-02344-s001.zip › ijms-1604603-supplementary.pdf]

Supplementary material

Urinary proteomic signature in acute decompensated heart failure: advances into molecular pathophysiology

Elisa Diaz-Riera[1,2]; Maisa García-Arguinzonis[1], Laura López[3], Xavier Garcia-Moll[3,4], Lina Badimon L[1,4,5#], Padro T[1,4#*]

1 Cardiovascular-Program ICCC; Research Institute – Hospital Santa Creu i Sant Pau, IIB-Sant Pau, 08041 Barcelona, Spain; ediazr@santpau.cat (ED-R); mgarciaar@santpau.cat (MG-A); lbadimon@santpau.cat (LB); tpadro@santpau.cat (TP)

2 Faculty of Medicine, Universtitat de Barcelona, 08036 Barcelona, Spain

3 Cardiology department, Hospital Santa Creu i Sant Pau, 08025 Barcelona, Spain; llopezl@santpau.cat (LL); xgarcia-moll@santpau.cat (XG-M)

4 Centro de Investigación Biomédica en Red Cardiovascular (CIBERCV) Instituto de Salud Carlos III, 28029 Madrid, Spain

5 Cardiovascular Research Chair, UAB, 08025 Barcelona, Spain

# *Equal contribution*

***Correspondence:**

Dr Teresa Padro

tpadro@santpau.cat
Tel.: +34935565886;
Fax: +34-935565559

Supplementary Table S1: Clinical characteristics and risk factors of all patients.

	All patients N=67	NRF N=35	RD N=32	P-value
**DEMOGRAPHIC CHARACTERISTICS**				
Female/male, n	22/45	10/25	12/20	0.603
Age, years	71.0 [65.0-77.0]	69.0 [58.0-75.0]	74.0 [69.5-77.5]	**0.008**
Weight, kg	73.0 [61.6-86.8]	70 [61.2-86.6]	77.2 [62.0-88.6]	0.543
**KIDNEY FUNCTION MARKERS**				
Creatinine, μmol/L	105.0 [78.0-147.0]	78.0 [67.0-97.0]	147.0 [122.5-194.0]	**<0.001**
Glomerular filtration (MDRD-4)[a]	61.0 [40.9-83.3]	83.0 [68.9-99.0]	39.7 [31.3-45.0]	**<0.001**
Urea, mmol/L	10.2 [6.8-16.1]	7.0 [5.8-9.2]	16.5 [13.0-22.9]	**<0.001**
**CARDIAC FUNCTION MARKERS**				
NT-proBNP, ng/L	4.0 [2.3-8.6]	3.2 [1.9-6.4]	4.5 [2.8-14.7]	**0.026**
Left ventricular ejection fraction (LVEF), %	45.0 [33.0-58.0]	38.0 [33.0-57.0]	51.0 [35.5-59.5]	0.225
Preserved LVEF, N (%)	28 (42)	11 (31)	17 (53)	
Reduced LVEF, N (%)	27 (40)	18 (52)	9 (28)	0.125
Mid-range LVEF, N (%)	12 (18)	6 (17)	6 (19)	
Atrial fibrillation, N (%)	32 (48)	16 (47)	16 (50)	>0.999
Cardiovascular disease, N (%)	23 (34)	11 (31)	12 (38)	0.618
**OTHER BIOCHEMICAL MARKERS**				
Haemoglobin, g/L	122 [101-138]	128 [114-142]	110 [95-124]	**0.004**
**RISK FACTORS; N (%)**				
Active smoking	10 (15)	8 (23)	2 (6)	0.086
Hypertension	49 (74)	21 (62)	28 (88)	**0.024**
Pulmonary hypertension	16 (24)	8 (23)	8 (25)	>0.999
Diabetes mellitus type 2	30 (45)	11 (32)	19 (59)	**0.047**
Dyslipidaemia	46 (70)	20 (59)	26 (81)	0.063
**BACKGROUND MEDICATION; N (%)**				
Diuretics	49 (73)	21 (60)	28 (88)	**0.014**
Statins	43 (64)	18 (51)	25 (78)	**0.040**
Anticoagulants	27 (40)	14 (40)	13 (41)	>0.999
Antiplatelet agents	38 (57)	19 (54)	19 (59)	0.806
Beta-blockers	46 (69)	21 (60)	25 (78)	0.124
Antiarrhythmic agents	7 (10)	4 (11)	3 (9)	>0.999
Antidiabetics	27 (40)	8 (26)	19 (59)	**0.003**
Insulin	12 (18)	2 (6)	10 (31)	**0.010**
Oral antidiabetic agents	22 (33)	8 (23)	14 (44)	0.117
ACE inhibitor/ARB	43 (66)	26 (74)	17 (57)	0.189

[a]MDRD-4 levels expressed in mL/min/1.73m^2; Quantitative values were given in median [Q1-Q3]. P values of categorical variables were calculated with Fisher exact test, except for LVEF where χ^2 was used. P values of numerical data were calculated with Mann-Whitney, except for LVEF where Kruskall-Wallis was used. Diuretics: hydrochlorothiazide, furosemide, eplerenone, and spironolactone. Statins: atorvastatin, pravastatin, simvastatin, ezetimibe. Anticoagulants: warfarin, acenocumarol, bemiparin, heparin, dabigatran, rivaroxaban, edoxaban, and apixaban. Antiplatelet agents: acetylsalicylic acid and clopidogrel. Beta-blockers: bisoprolol and carvedilol. Antiarrhythmic agents: amiodarone. Oral antidiabetic agents: metformin and repaglinide. Angiotensin-converting enzyme inhibitors (ACEI) include: captopril, enalapril, and ramipril. Angiotensin receptor blockers (ARB): losartan, olmesartan, and valsartan.

Supplementary Table S2: Clinical characteristics and risk factors of discovery phase patients.

	2DE-MS group N=17	All patients N=67	P value
**DEMOGRAPHIC CHARACTERISTICS**			
Female/male, n	3/14	22/45	0.565
Age, years	72 [69-76]	71 [65-77]	0.624
Weight, kg	76 [66-87]	73 [62-89]	0.583
**KIDNEY FUNCTION MARKERS**			
Creatinine, µmol/L	101 [73-131]	105.0 [78.0-147.0]	0.693
Glomerular filtration (MDRD-4)[a]	68 [43.2-81.7]	61.0 [40.9-83.3]	0.476
Urea, mmol/L	9.0 [5.8-13.1]	10.2 [6.8-16.1]	0.367
**CARDIAC FUNCTION MARKERS**			
NT-proBNP, µg/L	2.4 [1.7-4.6]	4.0 [2.3-8.6]	0.160
Left ventricular ejection fraction (LVEF), %	48 [33-56]	45 [33-58]	0.738
Preserved LVEF >50%, N (%)	8 (47)	28 (42)	
Mildly reduced LVEF 40-49%, N (%)	3 (18)	12 (18)	0.916
Reduced LVEF <40%, N (%)	6 (35)	27 (40)	
**OTHER BIOCHEMICAL MARKERS**			
Haemoglobin, g/L	122 [106-139]	122 [101-138]	0.889
**MEDICAL HISTORY; N (%)**			
Atrial fibrillation	12 (71)	32 (48)	0.283
Cardiovascular disease	4 (24)	23 (34)	0.563
Active smoking	3 (5)	10 (15)	0.721
Hypertension	12 (71)	49 (74)	>0.999
Pulmonary hypertension	6 (35)	16 (24)	0.364
Diabetes mellitus type 2	6 (35)	30 (45)	0.586
Dyslipidaemia	11 (65)	46 (70)	>0.999
**BACKGROUND MEDICATION; N (%)**			
Diuretics	11 (65)	49 (73)	0.253
Statins	9 (53)	43 (64)	0.415
Anticoagulants	10 (59)	27 (40)	0.415
Antiplatelet agents	7 (41)	38 (57)	0.286
Beta-blockers	11 (65)	46 (69)	0.777
Antiarrhythmic agents	0 (0)	7 (10)	0.335
Antidiabetics	3 (24)	27 (40)	0.265
ACE inhibitor/ARB	13 (76)	43 (66)	0.562

[a]MDRD-4 levels expressed in mL/min/1.73m^2; Quantitative values were given in median [Q1-Q3]; 2DE-MS group refers to a subgroup of patients used for discovery phase of 2D electrophoresis coupled with mass spectrometry. P values of categorical were calculated with Fisher exact test, except for LVEF where χ^2 was used. P values of numerical data were calculated with Mann-Whitney, except for LVEF where Kruskall-Wallis was used. Diuretics: hydrochlorothiazide, furosemide, eplerenone, and spironolactone. Statins: atorvastatin, pravastatin, simvastatin, ezetimibe. Anticoagulants: warfarin, acenocumarol, bemiparin, heparin, dabigatran, rivaroxaban, edoxaban, and apixaban. Antiplatelet agents: acetylsalicylic acid and clopidogrel. Beta-blockers: bisoprolol and carvedilol. Antiarrhythmic agents: amiodarone. Oral antidiabetic agents: metformin and repaglinide. Angiotensin-converting enzyme (ACE) inhibitors include: captopril, enalapril, and ramipril. Angiotensin receptor blockers (ARB): losartan, olmesartan, and valsartan.

Supplementary Table S3: Extended data from table 1 regarding mass spectrometry characteristics of identified proteins in urine of ADHF patients.

Gel-ID	Protein name	Gene name	Swiss Prot number	Theoretical pI	Experimental pI	Theoretical MW (KDa)	Experimental MW (KDa)	MS or MS/MS	MASCOT Score	Coverage
1	Lysosomal acid phosphatase	*ACP2*	P11117	5.80	5.80	46.7	45.5	MS	63	7
2	Pancreatic α-amylase	*AMY2A*	P04746	6.45	6.45	57.7	51.6	MS/MS	61	-
3	Annexin A10	*ANXA10*	Q9UJ72	5.13	5.20	37.3	35.2	MS	60	19
4	Arylsulphatase A	*ARSA*	P15289	5.57	5.50	53.6	49.6	MS	67	10
5	Zinc-α-2-glycoprotein	*AZGP1*	P25311	5.58	4.8-5.1	34.3	41.6-43.5	MS	66	11
6	Complement C3	*C3*	P01024	6.00	6.75	187.1	54.8	MS	62	3
7	Carbonic anhydrase 1	*CA1*	P00915	6.63	6.70	28.9	30.1	MS/MS	71	-
8	Endosialin	*CD248*	Q9HCU0	5.14	4.70	80.9	44.6-45.6	MS/MS	54	
9	CD59 glycoprotein	*CD59*	P13987	5.18	4.90	14.2	22.7	MS/MS	82	-
10	Cathepsin D	*CTSD*	P07339	5.60	5.40	44.6	31.2	MS/MS	58	-
11	Fibrinogen β-chain	*FGB*	P02675	7.95	4.90	55.9	18.7-19.8	MS/MS	55	
12	Fibrinogen γ-chain	*FGG*	P02679	5.24	5.30-5.35	55.5	48.0-48.2	MS/MS	55	-
13	Vitamin D binding protein	*GC*	P02774	5.22	5.20	52.9	50.5	MS	94	17
14	Hemopexin	*HPX*	P02790	6.43	5.30-5.35	51.7	55.4	MS/MS	70	
15	Basement membrane-specific heparan sulphate proteoglycan core protein	*HSPG2*	P98160	6.03	5.40	468.8	24.9	MS/MS	102	-
16	Inter-α-trypsin inhibitor heavy chain H4	*ITIH4*	Q14624	6.00	4.9-5.1	103.4	36.6-37.2	MS	74	10
17	Kininogen-1	*KNG1*	P01042	6.23	4.7-4.9	72.0	50.5-53.3	MS	63	8
18	Vesicular integral-membrane protein VIP36	*LMAN2*	Q12907	6.06	5.20	40.2	35.2	MS/MS	60	-
19	Leucine-rich α-2-glycoprotein	*LRG1*	P02750	5.66	4.60	38.2	47.4	MS/MS	79	-
20	Retinol binding protein 4	*RBP4*	P02753	5.27	5.20	23.0	24.7-25.2	MS/MS	66	-
21	α-1-antitrypsin	*SERPINA1*	P01009	5.37	5.00-5.10	46.7	51.4-52.2	MS	130	19
22	Antithrombin III	*SERPINC1*	P01008	5.95	5.20	52.6	52.6	MS/MS	78	-
23	Serotransferrin	*TF*	P02787	6.70	6.00-6.40	77.1	56.5	MS	234	26
24	Trefoil factor 2	*TFF2*	Q03403	5.21	5.20	14.3	11.1	MS/MS	83	-
25	Transthyretin	*TTR*	P02766	5.31	5.30	15.9	15.9	MS/MS	61	-
26	Vitelline membrane outer layer protein 1 homolog	*VMO1*	Q7Z5L0	4.65	4.65	21.2	21.2	MS/MS	87	-

pI: isoelectric point; MW: molecular weight; MS: mass spectrometry.

Supplementary Table S4: Selected peptide information of proteins identified by MS/MS in urine of ADHF patients.

Gel-ID	Protein name	Gene name	Swiss Prot number	M/Z	Position	Sequence	Modification	Miss-cleavage
2	Pancreatic α-amylase	*AMY2A*	P04746	**1427.7**	307-318	ALVFVDNHDNQR	-	0
				1570.7	88-100	SGNEDEFRNMVTR	MSO: 97	1
7	Carbonic anhydrase 1	*CA1*	P00915	**985.4**	82-90	GGPFSDSYR	-	0
				970.6	161-169	VLDALQAIK	-	0
8	Endosialin	*CD248*	Q9HCU0	**1198.6**	93-101	QCQLQRPLR	Cys_CAM: 94	0
				1072.5	216-225	QPEGGVGWSR	-	0
9	CD59 glycoprotein	*CD59*	P13987	**1539.7**	67-78	FEHCNFNDVTTR	Cys_CAM: 70	0
10	Cathepsin D	*CTSD*	P07339	**1462.7**	393-403	YYTVFDRDNNR	-	1
11	Fibrinogen β-chain	*FGB*	P02675	**1032.6**	484-491	IRPFFPQQ	-	0
12	Fibrinogen γ-chain	*FGG*	P02679	**1034.5**	293-301	VGPEADKYR	-	1
				1194.5	32-40	DNCCILDER	Cys_CAM: 34, 35	0
				1545.8	418-432	LTIGEGQQHHLGGAK	-	0
14	Hemopexin	*HPX*	P02790	**1220.6**	92-102	NFPSPVDAAFR	-	0
				1268.7	209-219	FDPVRGEVPPR	-	1
				1684.9	209-222	FDPVRGEVPPRYPR	-	2
15	Basement membrane-specific	*HSPG2*	P98160	1666.8	4282-4295	LVSEDPINDGEWHR	-	0
	heparan sulphate proteoglycan			**1601.9**	4304-4318	RGSIQVDGEELVSGR	-	1
	core protein			2413.2	4358-4379	NLVLHSARPGAPPPQPLDLQ HR	-	0
18	Vesicular integral-membrane	*LMAN2*	Q12907	1343.7	208-218	NRDHDTFLAVR	-	1
	protein VIP36			**1073.5**	210-218	DHDTFLAVR	-	0
19	Leucine-rich α-2-glycoprotein	*LRG1*	P02750	989.55	251-260	VAAGAFQGLR	-	0
				**1152.6**	165-175	ALGHLDLSGNR	-	0
20	Retinol binding protein 4	*RBP4*	P02753	**1106.5**	29-37	VKENFDKAR	-	2
				1302.7	172-181	QRQEELCLAR	Cys_CAM: 178	1
				1675.8	185-198	LIVHNGYCDGRSER	Cys_CAM: 192	1
22	Antithrombin III	*SERPINC1*	P01008	**1674.8**	202-215	LQPLDFKENAEQSR	-	1
24	Trefoil factor 2	*TFF2*	Q03403	**1696.7**	90-104	NCGYPGISPEECASR	Cys_CAM: 91, 101	0
25	Transthyretin	*TTR*	P02766	**1394.7**	56-68	AADDTWEPFASGK	-	0
26	Vitelline membrane outer layer protein 1 homolog	*VMO1*	Q7Z5L0	**1217.7**	185-196	GLGDDTALNDAR	-	0

M/Z: mass to charge ratio peaks, in bold those with highest MASCOT scores; position of amino acids corresponding to each peak and its sequence; variable modifications accepted: carbamidomethyl (CAM) and methionine sulfoxide (MSO); miss-cleavage: up to two trypsin miss-cleavages accepted.

Supplementary Table S5: Biological processes and molecular functions of differential urinary proteins.

SwissProt number	Gene	Biological processes				Molecular functions		
		Cell function	Haemostatic & complement system	Inflammation & immune response	Metabolic processes	Binding	Catalytic activity	Transport
P11117	***ACP2***				●		●	
P04746	***AMY2A***				●	●	●	
Q9UJ72	***ANXA10***				●	●		
P15289	***ARSA***				●	●		
P25311	***AZGP1***	●	●					●
P01024	***C3***		●	●	●	●	●	
P00915	***CA1***		●			●	●	
Q9HCU0	***CD248***	●				●		
P13987	***CD59***		●			●		
P07339	***CTSD***				●		●	
P02675	***FGB***	●	●	●		●		
P02679	***FGG***	●	●			●		
P02774	***GC***				●	●		●
P02790	***HPX***				●	●		●
P98160	***HSPG2***	●		●	●	●		
Q14624	***ITIH4***			●			●	
P01042	***KNG1***		●	●		●	●	
Q12907	***LMAN2***				●	●		●
P02750	***LRG1***	●		●		●		
P02753	***RBP4***		●		●	●		●
P01009	***SERPINA1***		●	●		●	●	
P01008	***SERPINC1***		●	●		●	●	
P02787	***TF***		●	●		●		●
Q03403	***TFF2***		●			●		
P02766	***TTR***				●	●		●
Q7Z5L0	***VMO1***					●	●	

Cell function includes: angiogenesis, cell adhesion, differentiation, and migration.

Supplementary table S6: Urinary transthyretin levels in relation to patient clinical characteristics.

	ADHF patients		NRF		RD	
	N	Median [IQR]	N	Median [IQR]	N	Median [IQR]
**LVEF**						
Reduced (<40%)	27	12.3 [3.3-64.1]	18	12.3 [3.3-64.1][a]	9	12.0 [3.3-54.5][b]
Mildly reduced (40-49%)	12	15.0 [4.8-71.1]	6	9.7 [3.6-41.8]	6	19.6 [15.0-71.1]
Preserved (≥50%)	28	7.8 [3.7-64.4]	11	4.1 [1.6-5.0]	17	15.1 [7.2-70.7]
P value		*0.653*		*0.126*		*0.428*
**Hypertension**						
No	17	3.9 [2.8-64.1]	13	3.6 [2.4-64.1]	4	5.0 [3.3-70.7]
Yes	49	15.0 [5.0-64.4]	21	11.0 [4.2-43.0]	28	18.3 [7.6-71.1]
P value		*0.144*		*0.433*		*0.353*
**Dyslipidaemia**						
No	20	5.3 [3.3-54.5]	13	4.6 [3.0-40.5]	6	10.3 [5.0-54.5]
Yes	46	15.1 [4.3-67.1]	20	12.3 [2.2-44.2]	26	18.9 [7.6-72.3]
P value		*0.205*		*0.685*		*0.263*
**Diabetes mellitus type II**						
No	36	5.5 [3.3-44.2]	23	4.8 [3.3-41.8]	13	11.2 [5.0-54.5]
Yes	30	18.1 [7.6-71.1]	11	15.2 [1.6-45.0]	19	19.6 [7.6-85.2]
P value		*0.098*		*0.767*		*0.160*
**Cardiovascular disease**						
No	44	8.7 [3.4-43.4]	24	4.5 [2.4-35.1]	20	14.8 [5.0-64.4]
Yes	23	15.0 [5.3-97.3]	11	12.1 [2.8-64.1]	12	41.1 [7.2-128.3]
P value		*0.145*		*0.416*		*0.270*
**Atrial fibrillation**						
No	34	14.8 [4.8-64.4]	18	9.6 [3.3-64.1]	16	15.1 [7.2-64.4]
Yes	32	8.7 [3.3-54.5]	16	4.3 [2.2-41.8]	16	24.2 [3.7-84.8]
P value		*0.701*		*0.439*		*0.800*
**Pulmonary hypertension**						
No	51	11.2 [3.3-64.4]	27	5.2 [2.4-44.2]	24	18.3 [7.6-70.7]
Yes	16	12.3 [3.7-54.5]	8	8.2 [3.6-16.9]	8	15.0 [3.7-85.2]
P value		*0.893*		*0.962*		*0.811*

Urinary transthyretin levels in ng TTR/mg total protein. LVEF: left ventricular ejection fraction; NRF: ADHF patients with normal renal function at hospital admission; RD: ADHF patients with renal dysfunction at hospital admission; P values were calculated by Mann-Whitney test except for LVEF where Kruskall-Wallis test was used. Information of one patient was missing for hypertension, dyslipidaemia and diabetes mellitus type 2. [a,b]Comparison reduced vs preserved LVEF (Mann-Whitney), [a]P=0.049, [b]P=0.363.

Supplementary table S7: ROC (associated receiver operating characteristic) curve (AUC) analysis for determining power of urinary TTR and RBP4 levels in ADHF patients for GFR discrimination.

**Protein**	**AUC Area**	**Lower limit**	**Upper limit**	**P-value**
TTR	0.633	0.489	0.776	*0.070*
RBP4	0.742	0.614	0.870	*<0.001*
**TTR + RBP4**	0.826	0.705	0.947	*<0.0001*

TTR: transthyretin; RBP4: retinol binding protein 4, AUC: area under the curve.

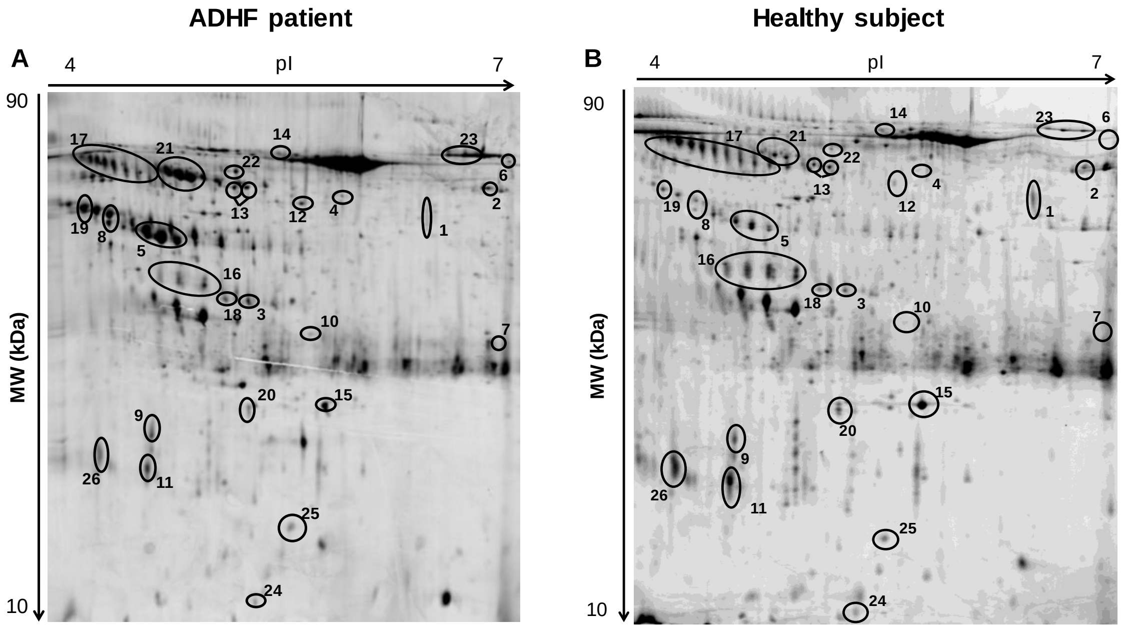

Supplementary Figure S1. 2DE-PAGE gels of urinary samples from a representative ADHF patient (A) and a representative healthy subject (B) in a pI range of 4-7 and 12% SDS-PAGE gels.

1	Lysosomal acid phosphatase	14	Hemopexin
2	Pancreatic α-amylase	15	Basement membrane-specific heparan sulphate proteoglycan core protein
3	Annexin A10	16	Inter-α-trypsin inhibitor heavy chain 4
4	Zinc α-2-glycoprotein	17	Kininogen 1
5	Arylsulphatase A	18	Vesicular integral-membrane protein VIP36
6	Complement C3	19	Leucine-rich α-2-glycoprotein
7	Carbonic anhydrase 1	20	Retinol binding protein 4
8	Endosialin	21	α-1-antitrypsin
9	CD59 glycoprotein	22	Antithrombin III
10	Cathepsin D	23	Serotransferrin
11	Fibrinogen β-chain	24	Trefoil factor 2
12	Fibrinogen γ-chain	25	Transthyretin
13	Vitamin D binding protein	26	Vitelline membrane outer layer protein 1 homolog

Protein	SwissProt number	Intensity change	MDRD-4				LVEF		
			T1	T2	T3		T1	T2	T3
FGB	P02675	down							
LMAN2	Q12907	down							
ANXA10	Q9UJ72	down							
RBP4	P02753	down							
HPX	P02790	up							
TTR	P02766	up							
GC	P02774	up							
ACP2	P11117	down							
AMY2A	P04746	down							
VMO1	Q7Z5L0	down							
ITIH4	Q14624	down							
SERPINA1	P01009	up							
KNG1	P01042	down							
FGG	P02679	up							
SERPINC1	P01008	up							
AZGP1	P25311	up							
HSPG2	P98160	down							
CD59	P13987	up							
C3	P01024	up							
CTSD	P07339	up							
ARSA	P15289	up							
TF	P02787	up							
CD248	Q9HCU0	up							
LRG1	P02750	up							
TFF2	Q03403	down							
CA1	P00915	up							

	Fold change
	1.5-2.0
	2.1-3.0
	3.1-5.0
	>5.1

	MDRD-4	LVEF
T1	33.7 [30.7-43.2]	33.0 [22.0-33.0]
T2	68.5 [62.5-71.7]	49.5 [47.0-55.0]
T3	97.8 [106.0-120.0]	60.0 [60.0-65.0]

Supplementary Figure S2. Fold change in urinary differential proteins according to renal function and left ventricular ejection fraction (LVEF) distributed by tertiles.

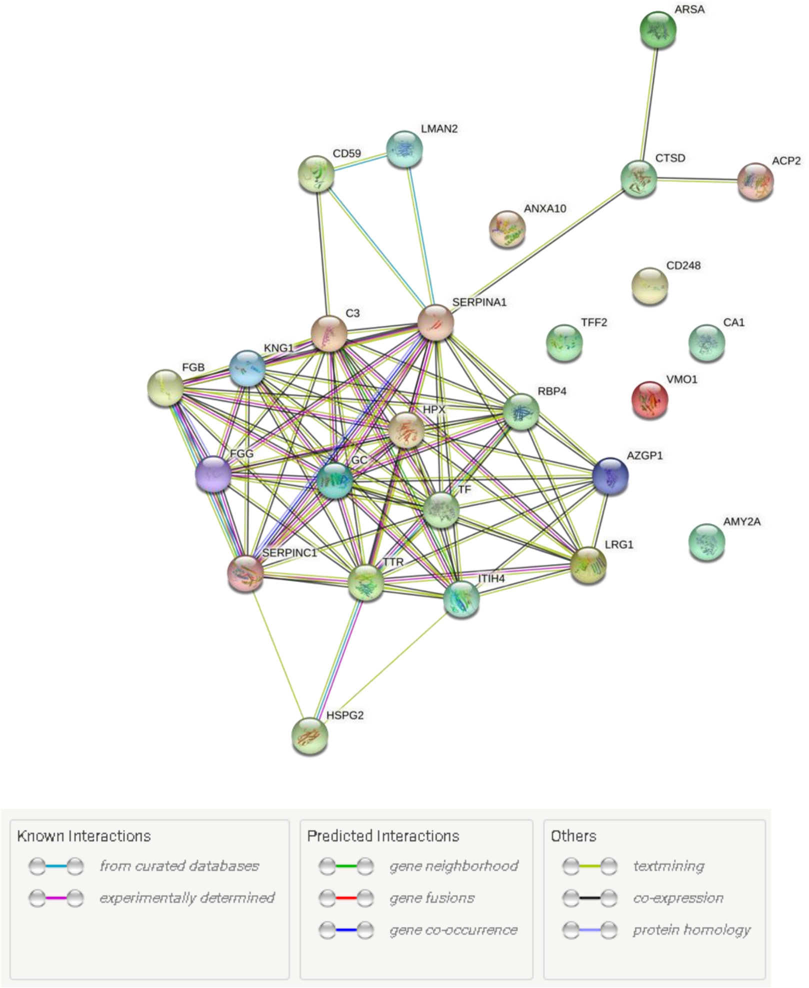

Supplementary Figure S3. Original STRING network showing the 26 differential proteins consistently detected in urine of ADHF patients and healthy subjects.

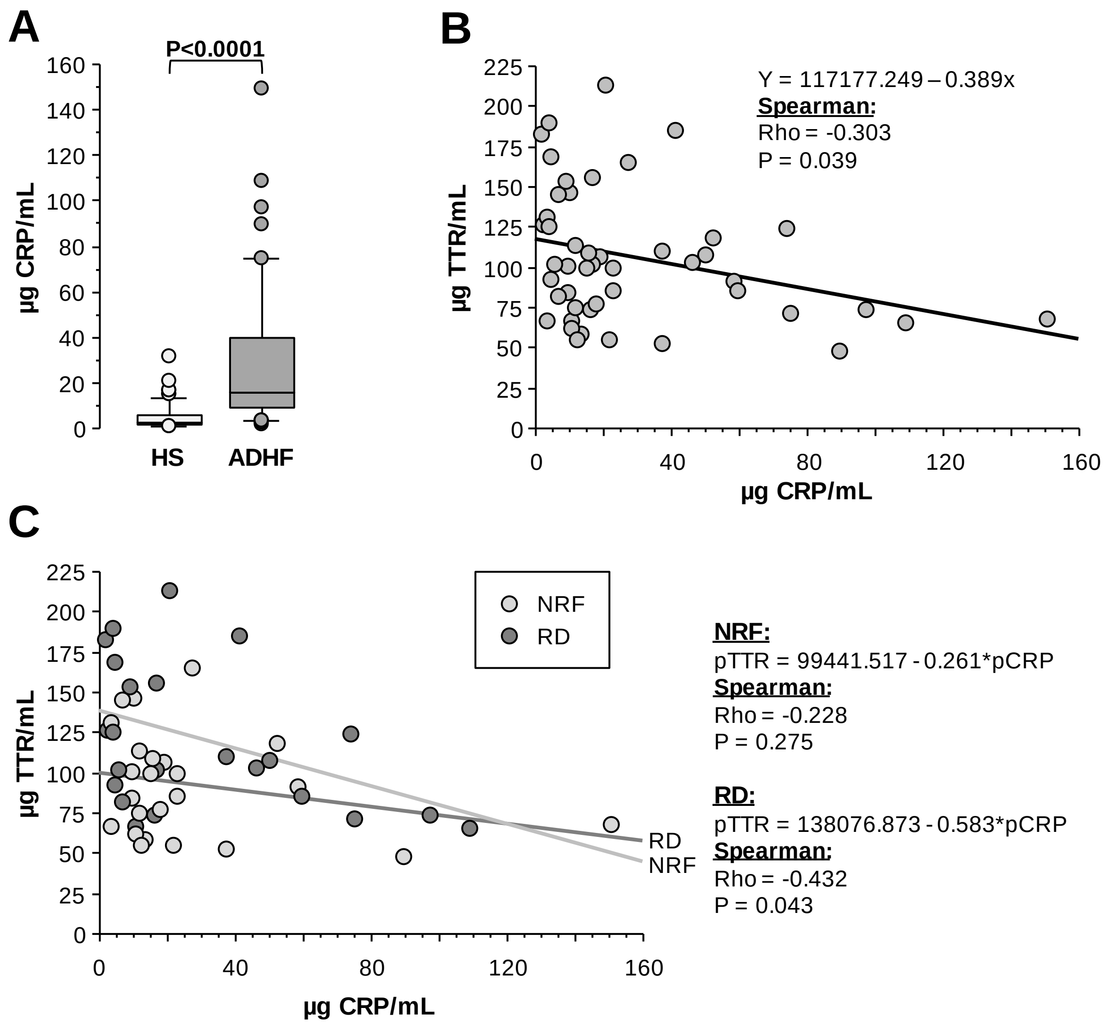

Supplementary Figure S4. Plasma C reactive protein (CRP) in ADHF patients at hospital admission. A) CRP levels of ADHF patients (N=67) and healthy subjects (HS). B) Significant correlation between CRP and TTR levels in ADHF patients. C) Regression lines of CRP and TTR correlations depending on kidney function, NRF in light grey and RD with dark grey.
